# Incidence and risk factors of postoperative acute pancreatitis after pancreaticoduodenectomy: a systematic review and meta-analysis

**DOI:** 10.3389/fsurg.2023.1150053

**Published:** 2023-05-09

**Authors:** Zhouyu Wu, Kezhen Zong, Baoyong Zhou, Kunli Yin, Anlan Zhang, Ming Li

**Affiliations:** Department of Hepatobiliary Surgery, The First Affiliated Hospital of Chongqing Medical University, Chongqing, China

**Keywords:** postoperative acute pancreatitis, pancreaticoduodenectomy, risk factors, systematic review, meta-analysis

## Abstract

**Background:**

Postoperative acute pancreatitis (POAP) is a specific complication after pancreatectomy. The acute inflammatory response of the residual pancreas may affect the healing of pancreatoenteric anastomoses, leading to postoperative pancreatic fistulas (POPFs), abdominal infections, and even progressive systemic reactions, conditions that negatively affect patients' prognoses and can cause death. However, to the best of our knowledge, no systematic reviews or meta-analytic studies have assessed the incidence and risk factors of POAP after pancreaticoduodenectomy (PD).

**Method:**

We searched PubMed, Web of Science, Embase, and Cochrane Library databases for relevant literature describing the outcomes of POAP after PD until November 25, 2022, and we used the Newcastle–Ottawa Scale to assess the quality of the studies. Next, we pooled the incidence of POAP and the odds ratios (ORs) and 95% confidence intervals (CIs) of the risk factors using a random-effect meta-analysis. *I*^2^ tests were used to assess heterogeneity between the studies.

**Results:**

We analyzed data from 7,164 patients after PD from 23 articles that met the inclusion criteria for this study. The subgroup results of the meta-analysis by different POAP diagnostic criteria showed that the incidences of POAP were 15% (95% CI, 5–38) in the International Study Group for Pancreatic Surgery group, 51% (95% CI, 42–60) in the Connor group, 7% (95% CI, 2–24) in the Atlanta group, and 5% (95% CI, 2–14) in the unclear group. Being a woman [OR (1.37, 95% CI, 1.06–1.77)] or having a soft pancreatic texture [OR (2.56, 95% CI, 1.70–3.86)] were risk factors of POAP after PD.

**Conclusion:**

The results showed that POAP was common after PD, and its incidence varied widely according to different definitions. Large-scale reports are still needed, and surgeons should remain aware of this complication.

**Systematic Review Registration:**

identifier: CRD42022375124.

## Introduction

Pancreaticoduodenectomy (PD) is a common surgical procedure for treating pancreatic head cancers and periampullary tumors (1). The procedure is one of the most complicated operations in hepatobiliary and pancreatic surgery, involving the removal of the pancreas, duodenum, and biliary tract and the reconstruction of the digestive tract (2). Improvements in surgical technique, equipment progress, and perioperative management have greatly reduced the mortality rate of PD to less than 3%, but its complication rate remains high at 30%. Complications increase treatment costs, prolong the length of hospital stay, and the risk of death (3). Delayed gastric emptying (DGE), pancreatic fistulas, hemorrhage and others are common postoperative complications of PD.

Postoperative acute pancreatitis (POAP) is commonly seen after operations involving the pancreas and its surrounding tissues and organs, such as PD, central pancreatectomy, and distal pancreatectomy (4, 5). However, POAP can also occur after heart or spinal surgical procedures (6, 7). The equally severe post-ERCP pancreatitis can also lead to local or systemic complications and even organ failure. The local acute inflammation of the pancreas may slow down the healing of pancreato-enteric anastomoses and residual pancreatic necroses, resulting in pancreatic fistulas, infections, and the need for secondary interventions (8–10).

This systematic review and meta-analysis aimed to provide an understanding of the frequency of POAP after PD and to explore the odds ratio and risk factors of POAP.

## Methods

We followed the PRISMA guidelines to design and implement this systematic review and meta-analysis (11, 12). We registered the study protocol in the PROSPERO database (CRD42022375124).

### Eligibility criteria

Considered for inclusion were observational studies of adults who underwent open or laparoscopic pancreaticoduodenectomy, including Whipple procedure, subtotal gastric preserved pancreaticoduodenectomy (SSPPD), and PD with pylorus preservation (PPPD), and reported the number or prevalence of POAP cases. We excluded case reports, case series, letters, reviews, and conference abstracts from our analysis.

### Search strategy

We searched PubMed, Web of Science, Embase, and Cochrane Library databases for relevant literature reporting the outcomes of POAP after PD from inception to November 25, 2022. We used Medical Subject Headings (MeSH) to search PubMed, Cochrane Library, and Web of Science, and Emtree terms were used to search Embase. We also scanned through citations and references to identify additional records. Our search was limited to articles in English and on human subjects.

We used the MeSH terms “Pancreaticoduodenectomy” and “Pancreatitis” for our PubMed search. The full search strategy is presented in the Supplementary Materials.

### Study selection

Two authors (ZW and KZ) independently screened the collected references for articles meeting our criteria. They read the headlines and abstracts to initially rule out all non-conforming studies. After the initial screening, the two authors performed full-text analyses. They read the articles and selected those to be included in our meta-analysis. Disagreements on the final inclusion of articles and the exclusion of duplicate studies were resolved through consultation between the two independent authors.

### Data extraction

The data extracted for each study included the name of the first author, country, enrollment period, sample size, age, gender, POAP diagnostic criteria applied, and the number of cases of POAP. We also extracted ORs and 95% CIs for the risk factor variables.

### Terminology and definitions

The Connor criteria (9), Atlanta definitions (13), and International Study Group for Pancreatic Surgery (ISGPS) definition (8) have been commonly used for diagnosing POAP. The Connor criteria defines POAP as the presence of urinary trypsinogen-2 levels higher than 50 µg/l or serum amylase levels higher than the normal upper limit on postoperative days (PODs) 0 or 1 (9).

The ISGPS defines POAP as an acute inflammatory response in the remnant pancreas early after partial pancreatectomy and serum amylase activity consistently above the normal upper limit for at least 48 h postoperatively, in addition to the presence of radiologic features and disease-related management changes (8). Finally, the Atlanta POAP classification and definition requires the presence of two of the following three features: abdominal pain consistent with acute pancreatitis; serum lipase or amylase activity at least three times higher than the normal upper limit; and radiologic features of acute pancreatitis (13).

### Study quality assessment

Two authors (ZW and KZ) independently assessed the quality of the studies included in the final analysis following the Newcastle–Ottawa Scale (NOS) guidelines. A third author was called to decide upon disagreements. The NOS score ranges between 7 and 9 for good quality studies, between 4 and 6 for moderate quality studies, and is lower than 4 for poor quality studies.

### Statistical analysis

We handled and analyzed all data using the R 4.1.1 software. Before using the “metaprop” function to combine incidence rates, we tested the original rate and the transformed rates for normality (sample rate estimation methods are as follows: “PRAW”, “PLN”, “ PLOGIT”, “PAS”, and “PFT”), and selected the transformation with the largest *p*-value of the test result to estimate rate. Odds ratios (ORs) and 95% confidence intervals (CIs) of risk factors were combined using the “metagen” function. We included studies with only crude ORs after calculating the adjusted ORs using the “calcOddsRatio” function. We assessed heterogeneity between studies using *I*^2^ tests. An *I*^2^ value higher than 50% indicated significant heterogeneity among studies and the need to pool the data using a random-effects model. We performed subgroup analyses by POAP diagnostic criteria. Sensitivity analyses were conducted using the “metainf” function. Finally, we used funnel plots and conducted an Egger's test to measure the risk of publication bias. We considered *p*-values < 0.05 as statistically significant.

## Results

### Study selection

**Figure 1:**
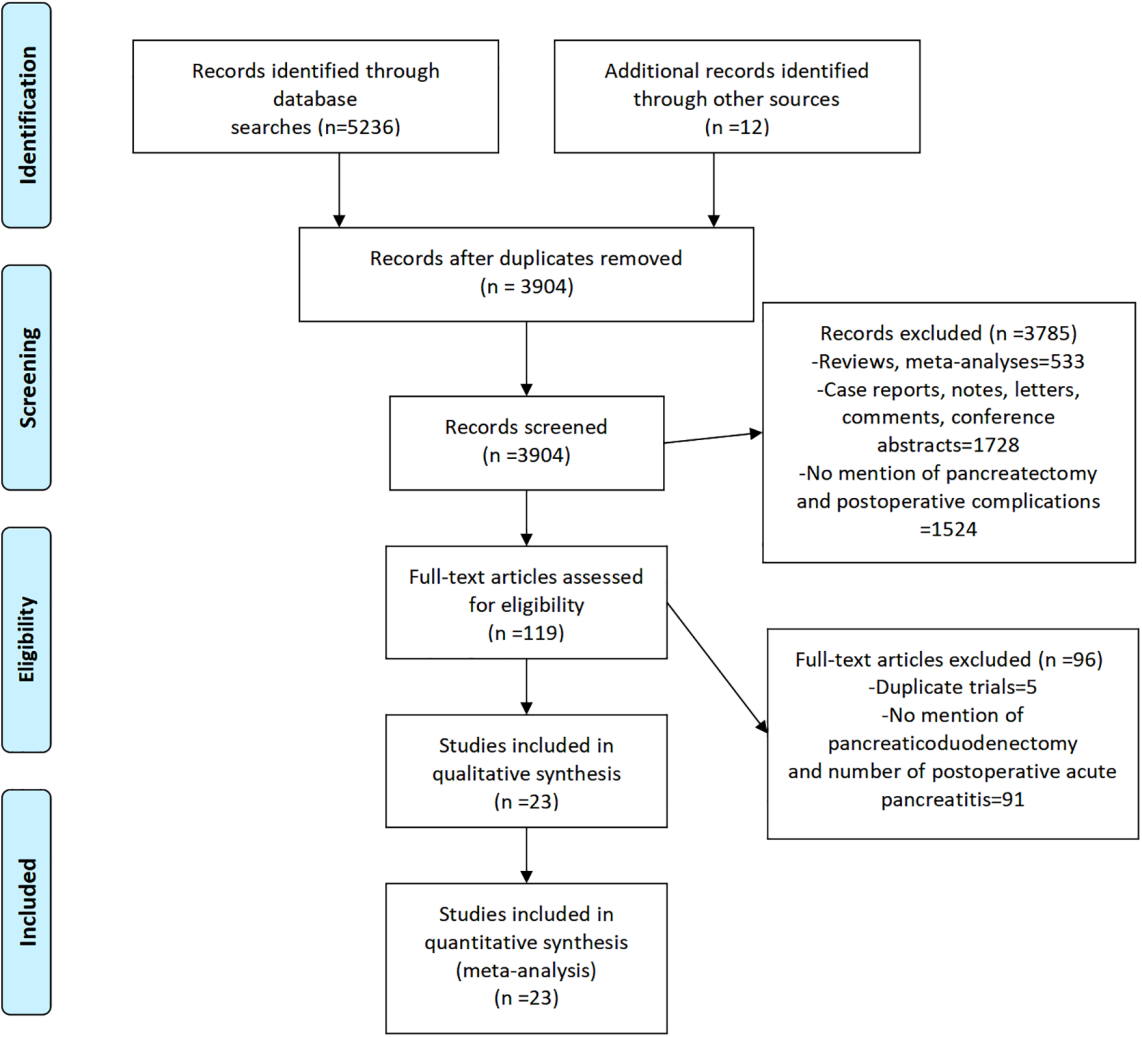
PRISMA flowchart (11) of the articles selected for inclusion in the meta-analysis.

Our search of PubMed, Cochrane Library, Web of Science, and Embase yielded 5,236 articles, and we manually retrieved an additional 12 articles for a total of 5,248 articles. We removed 1,344 duplicate articles and 3,785 nonconforming articles (case reports, case series, letters, reviews, and conference abstracts). Of the remaining 119 full-text articles, we excluded 96 because they were duplicated trials, failed to separately describe PD complications, or failed to describe POAP cases. Finally, we included data from 23 articles in this systematic review (Figure 1).

### Characteristics of included studies

Table 1 shows the main characteristics of the patients whose data were included in this study. We analyzed data from 7,164 pancreaticoduodenectomies, including 2,344 patients with POAP. Most of the studies included in this review were conducted in Europe (*n* = 11) and Asia (*n* = 8), with a few studies conducted in the United States (*n* = 1), Oceania (*n* = 1), and Africa (*n* = 1). There were 18 articles with definite POAP diagnostic criteria, while the other five studies did not specify the diagnostic criteria (unclear group).

**Table 1:** Characteristics of the studies included in the systematic review and meta-analysis.

Reference	Country	Enrollment period	All patients, *n*	Age,mean (SD)/median (range), years	Gender, M/F	BMI, mean (SD)/median (range), kg/m^2^	Diagnostic criteria of POAP	POAP, *n*	NOS score
Wu et al. (14)	China	2019–2021	286	62 (55–69)	176/110	NA	ISGPS	150	7
Murakawa et al. (15)	Japan	2013–2019	207	NA	NA	NA	Connor	121	8
Ikenaga et al. (42)	Japan	2015–2019	247	67 (59–73)	151/96	NA	ISGPS	9	8
Chen et al. (16)	China	2020–2021	716	63 (55–69)	423/293	22.8 (20.8–24.8)	ISGPS	152	7
Bonsdorff et al. (17)	Finland	2013–2020	508	68 (61–73)	277/231	25.5 (23.0–28.1)	Connor	202	7
Bannone et al. (18)	Italy	2016–2020	852	65 (56–72)	463/389	24 (22–26)	ISGPS	64	7
Yoo et al. (19)	South Korea	2015–2017	246	63.0 ± 9.2	152/94	24.3 ± 3.1	Connor	191	8
Paik et al. (20)	South Korea	2009–2019	163	63.4 ± 11.1	96/67	23.5 ± 3.7	Atlanta	41	6
Doussot et al. (21)	France	2020–2020	30	NA	23/7	NA	Connor	9	7
Ausania et al. (22)	Spain	2012–2018	62	NA	37/25	NA	Connor	27	6
Partelli et al. (23)	Italy	2015–2018	610	NA	308/302	NA	Connor	250	7
Chen et al. (24)	China	2010–2018	1,465	62 (54–68)	583/883	NA	Connor	770	8
Walsh et al. (25)	America	2001–2016	44	64.3 ± 14	14/30	NA	Unclear	1	7
Birgin et al. (26)	Germany	2009–2015	190	68 (59–74)	108/82	25 (23–28)	Connor	100	8
Nahm et al. (27)	Australia	2016–2017	35	67 (32–85)	18/17	NA	Connor	20	7
Kühlbrey et al. (28)	Germany	2001–2014	561	NA	NA	NA	Atlanta	200	7
Shuo et al. (29)	China	2011–2015	30	54.09 ± 9.3	52/31	NA	Unclear	1	8
Renz et al. (30)	Germany	2002–2012	300	NA	156/144	NA	Atlanta	9	7
Joliat et al. (45)	Switzerland	2002–2012	245	65 (54–75)	147/98	24.1 (21.6–26.5)	Atlanta	2	7
Dalla Valle et al. (31)	Italy	2009–2014	98	67.12 ± 10.44	55/43	24.48 ± 3.86	Unclear	3	7
Weinberg et al. (32)	Australia	2006–2012	150	67 (15–84)	89/61	26 (18–42)	Atlanta	8	6
Makni et al. (33)	Tunisia	1998–2009	80	56 ± 12.0	46/34	NA	Unclear	4	6
Räty et al. (34)	Finland	NA	39	60.2 ± 15.8	25/14	NA	Unclear	10	7

### Meta-Analyses

#### POAP incidence

**Figure 2:**
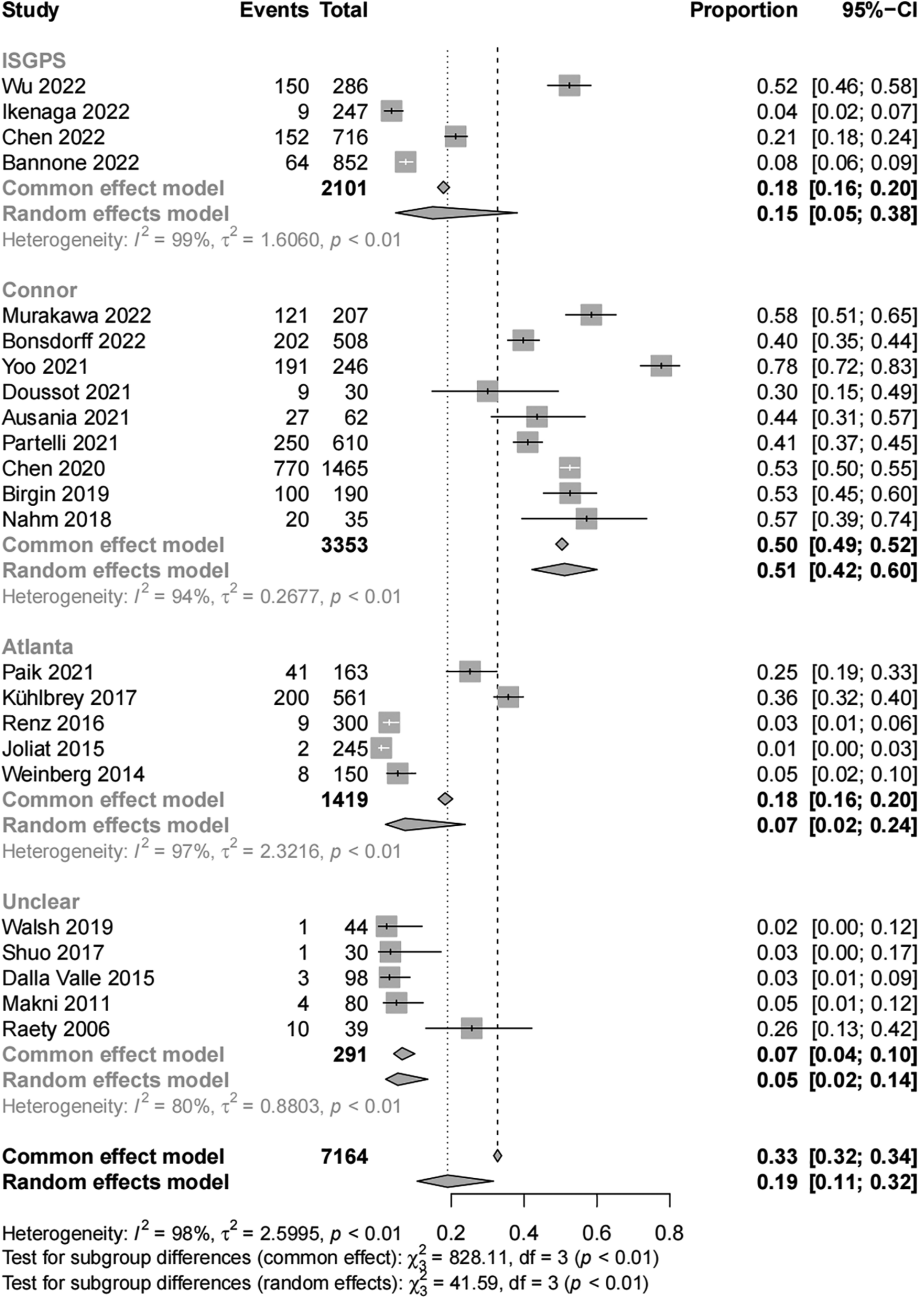
Subgroup analysis for incidence of POAP after PD according to diagnostic criteria.

The final results of the meta-analysis showed a 33% (95% CI, 32–34) incidence of POAP after PD, with significant heterogeneity among studies (*I*^2^ = 98%; *P* < 0.01). Subgroup analyses by POAP diagnostic criteria showed that the POAP incidences were 15% (95% CI, 5–38) in the ISGPS group, 51% (95% CI, 42–60) in the Connor group, 7% (95% CI, 2–24) in the Atlanta group, and 5% (95% CI, 2–14) in the unclear group (Figure 2).

#### Risk factor analysis for POAP

Results of our multifactorial analysis in some of the included studies showed that being a woman as well as having a small pancreatic duct diameter, soft pancreatic texture, and high body mass index (BMI) were all risk factors of POAP after PD (14, 16, 17, 26).

##### Sex

**Figure 3:**
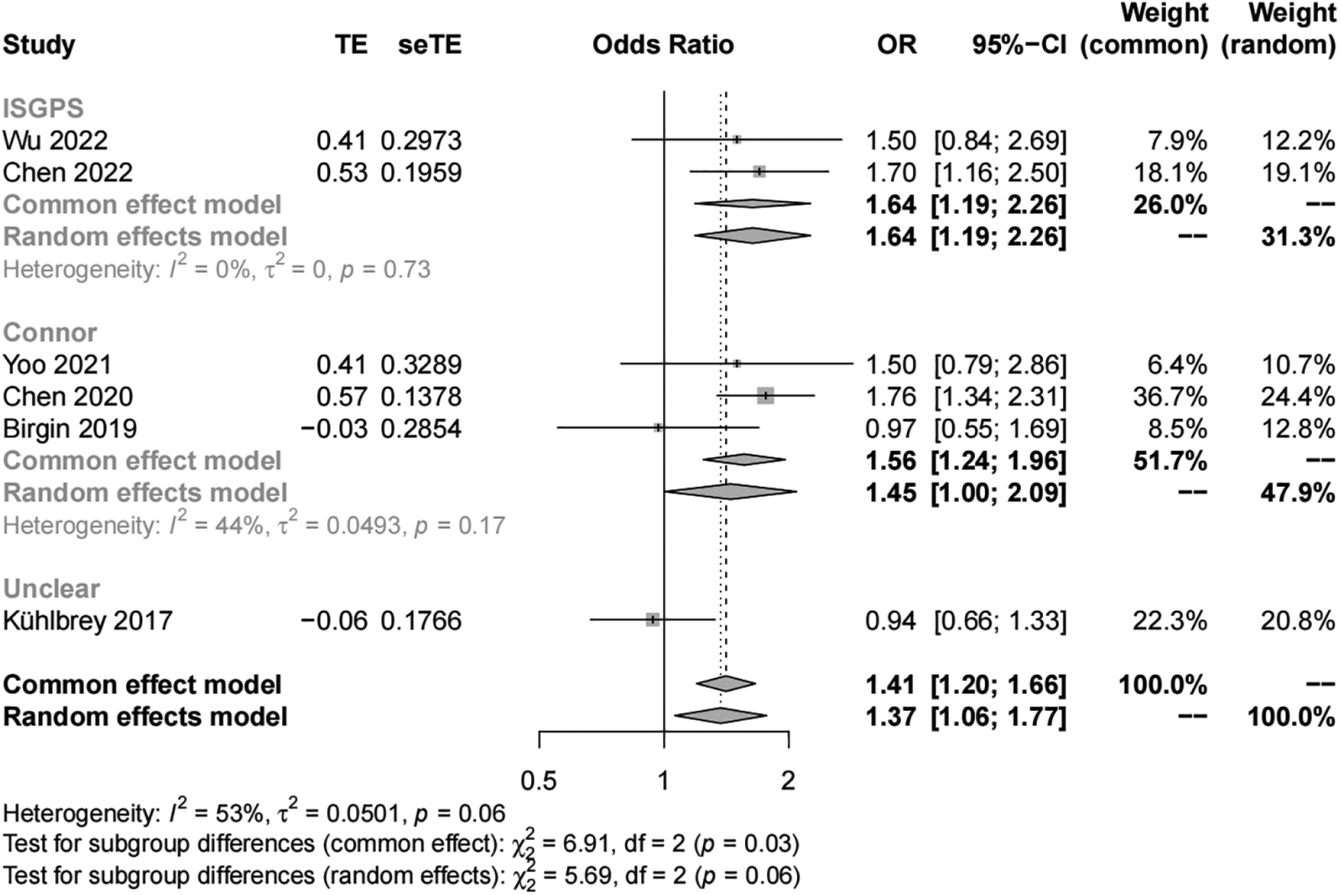
Subgroup analysis for sex according to POAP diagnostic criteria.

**Figure 4:**
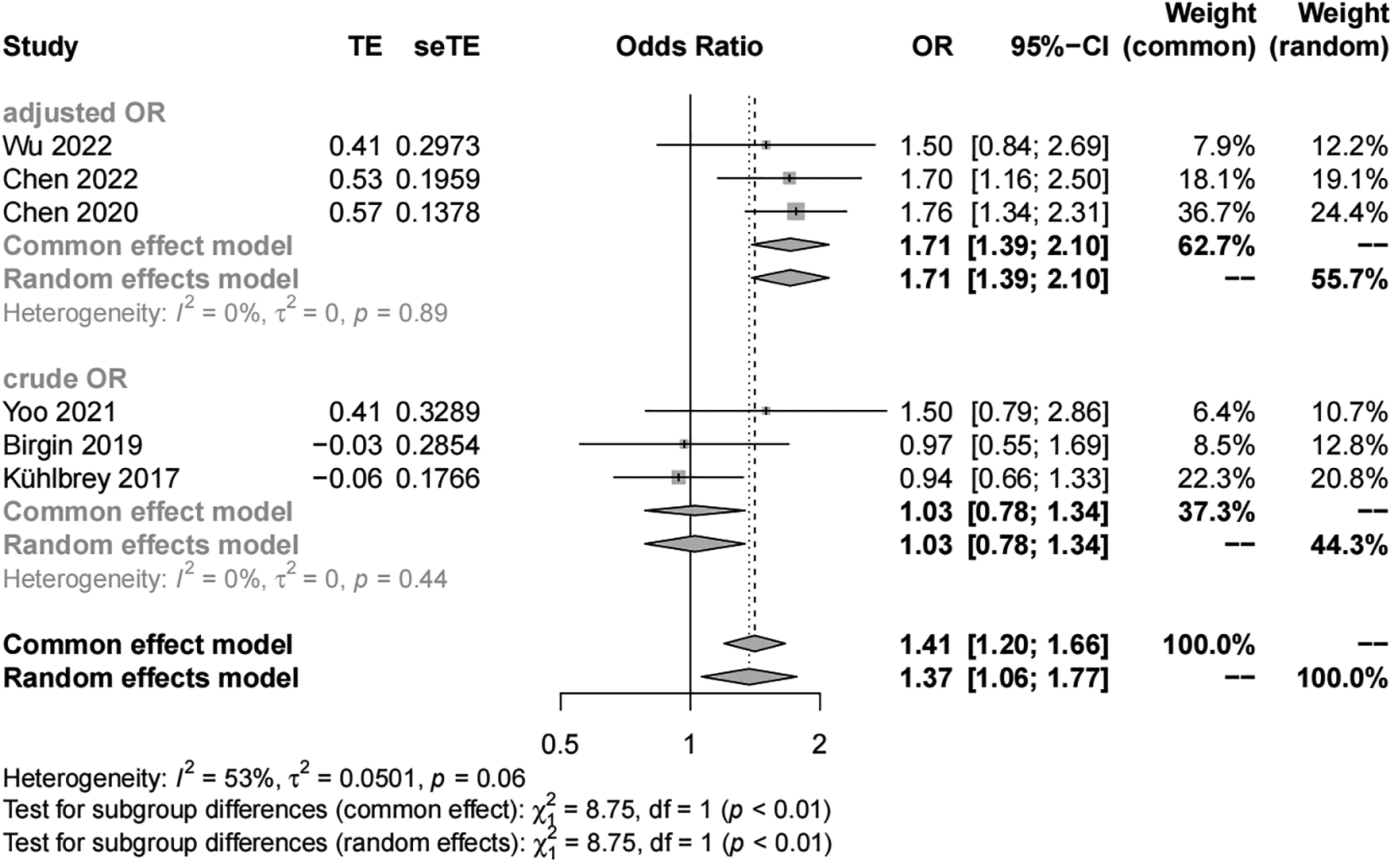
Subgroup analysis for sex according to adjusted/crude ORs.

We included six studies with 3,474 patients in the analysis of sex (female *vs*. male) as a risk factor. Our results showed significant study heterogeneity (*I*^2 ^= 53%). We pooled the data using a random effects model, and the results suggest that the risk of POAP after PD is approximately 1–2 times higher in women than in men (OR, 1.37; 95% CI, 1.06–1.77). Subgroup analyses were performed separately by POAP diagnostic criteria and adjusted/crude ORs. The results of the subgroup analyses by diagnostic criteria showed that the ORs were 1.64 (95% CI, 1.19–2.26) in the IGSPS group, 1.56 (95% CI, 1.24–1.96) in the Connor group, and 0.94 in the unclear group (95% CI, 0.66–1.33) (Figure 3). In addition, the results of subgroup analyses by adjusted/crude ORs showed that the ORs were 1.71 in the adjusted group (95% CI, 1.39–2.10) and 1.03 in the crude group (95% CI, 0.78–1.34) (Figure 4).

##### Pancreatic texture

**Figure 5:**
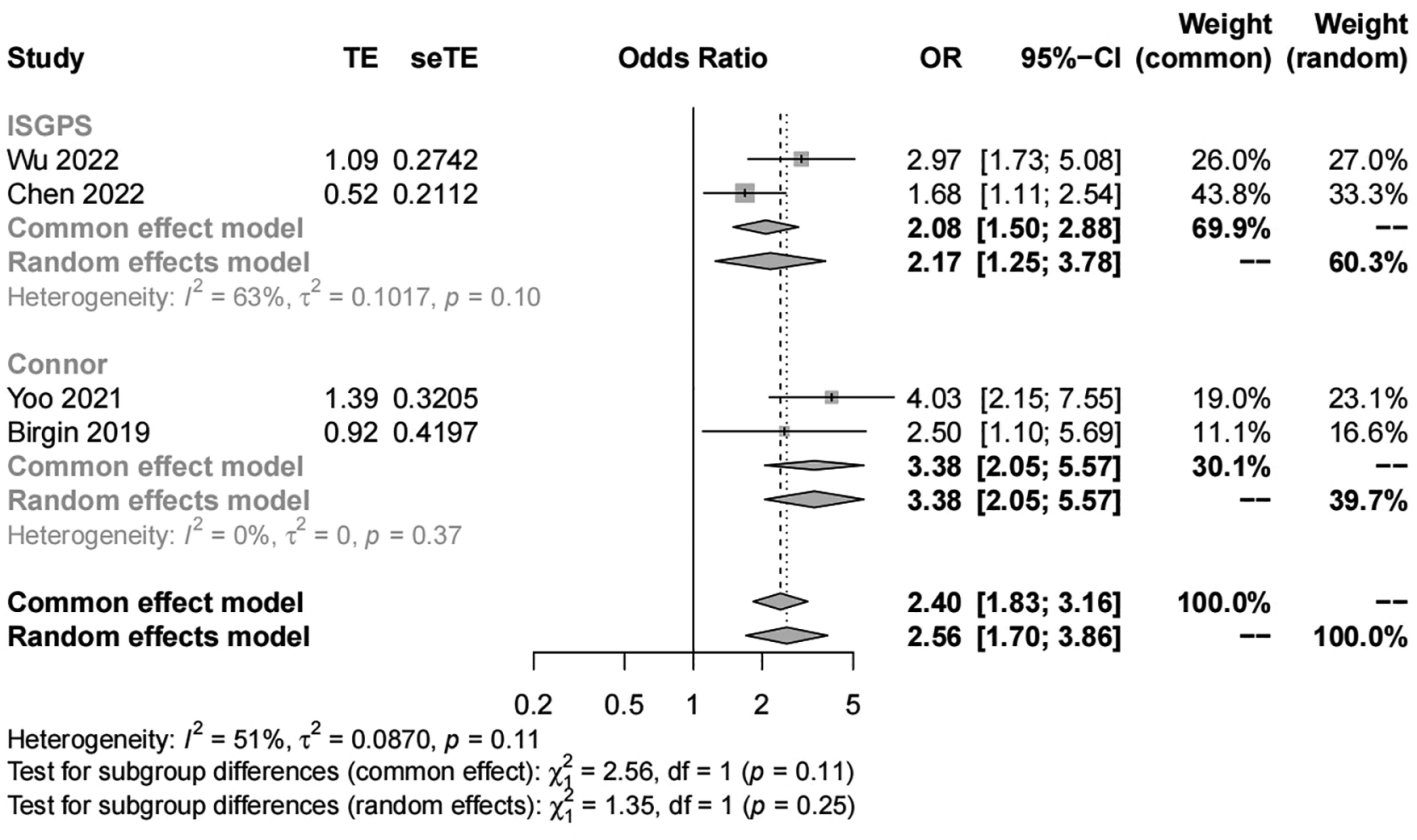
Subgroup analysis for pancreatic texture according to POAP diagnostic criteria.

**Figure 6:**
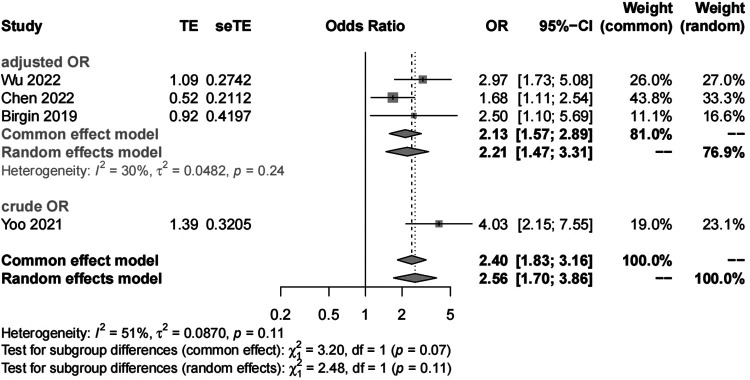
Subgroup analysis for pancreatic texture according to adjusted/crude ORs.

We included four studies with 1,419 patients in the analysis of pancreatic texture. Our results showed significant heterogeneity (*I*^2 ^= 51%) and suggested that patients with a soft pancreatic texture had a higher risk (OR, 2.56; 95% CI, 1.70–3.86) of POAP after PD than those with a hard pancreatic texture (Figures 5, 6). The results of subgroup analyses according to the diagnostic criteria revealed the following ORs: 2.17 for the IGSPS group (95% CI, 1.25–3.78) and 3.38 for the Connor group (95% CI, 2.05–5.57) (Figure 5). The results of subgroup analyses according to adjusted/crude ORs showed that the ORs were 2.13 for the adjusted group (95% CI, 1.57–2.89) and 3.38 for the crude group (95% CI, 2.05–5.57) (Figure 6).

### Sensitivity analysis

The results of our meta-analysis of pancreatic texture and total POAP incidence were not skewed by data from any individual study. However, in the meta-analysis of sex, the combined OR values changed after excluding individual studies (16, 24). See the Supplementary Material for details.

### Publication bias

**Figure 7:**
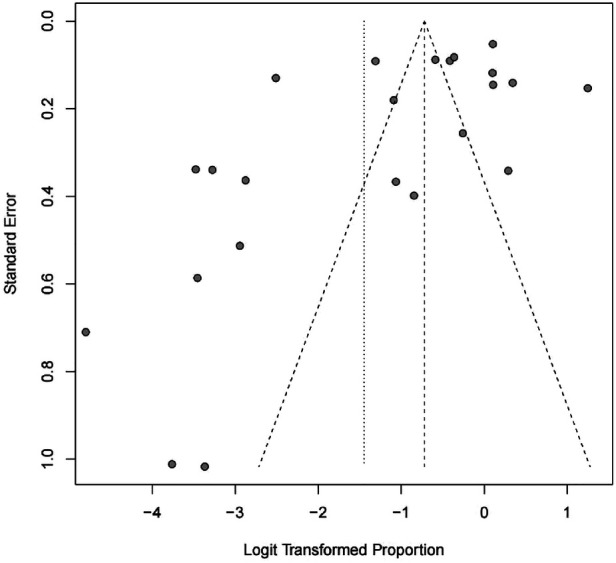
Funnel chart.

**Figure 8:**
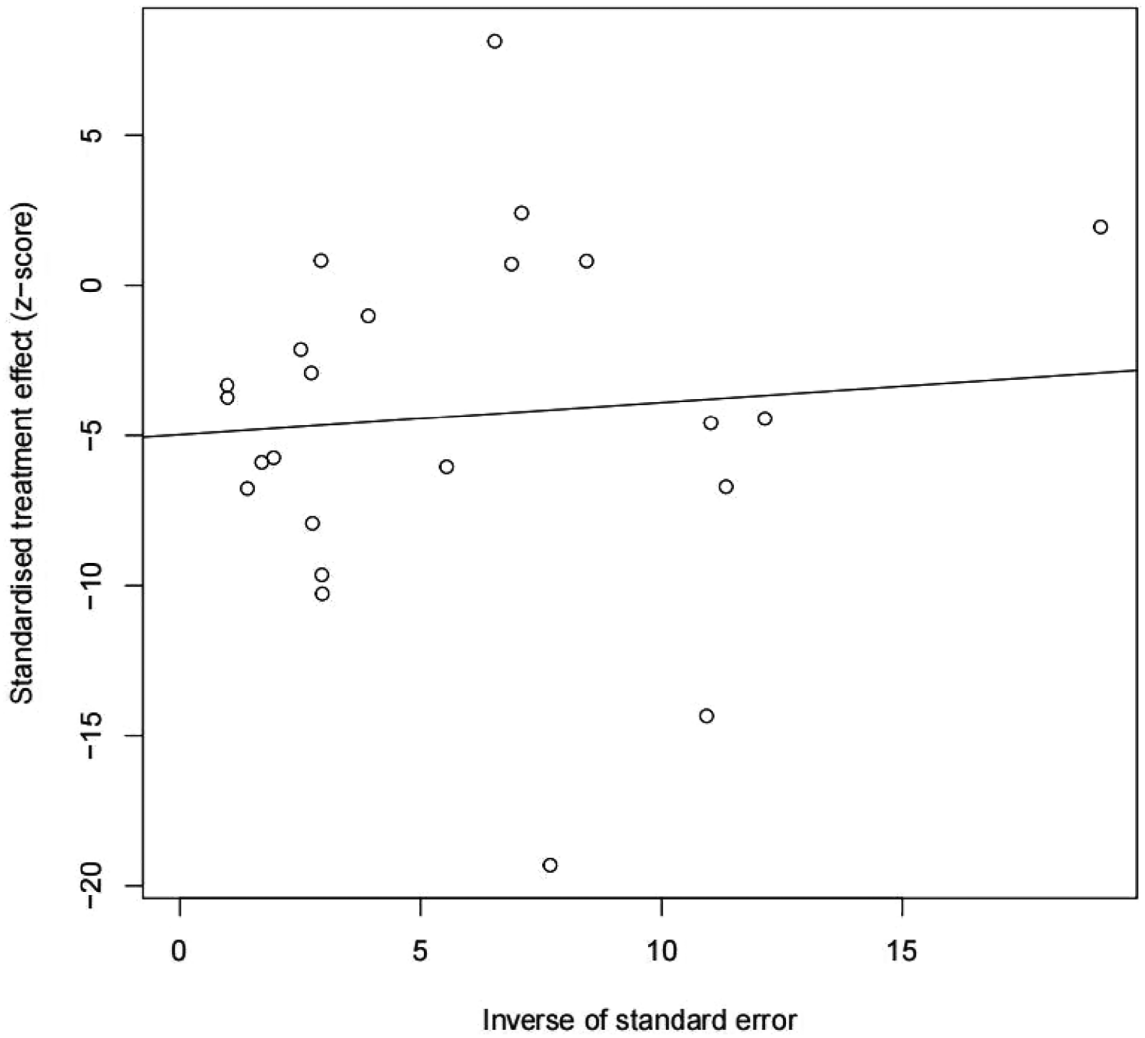
Egger funnel chart.

We found potential publication biases on the calculated incidence of POAP after PD. Figures 7, 8 show the funnel plots and Egger's test results (*P* = 0.024).

## Discussion

The main objective of this systematic review was to analyze the incidence of POAP after PD. This study included data from 23 studies, including 12 countries and 7,164 patients. The diagnostic criteria for POAP varied among the studies included, and the main diagnostic criteria were those from the ISGPS, the Connor criteria, and the Atlanta definition. According to the results of our meta-analysis, the overall incidence of POAP after PD was high (33%). The lack of standard POAP diagnostic criteria may have led to differences and errors in the POAP incidence calculated in the individual studies. In studies with clear diagnostic criteria, the POAP incidences were 15%, 51%, and 7% in those using the ISGPS definition, Connor criteria, and Atlanta definition, respectively. The lack of need for radiologic features of acute pancreatitis to diagnose POAP in the Connor criteria group may have caused the incidence of POAP to be significantly higher in this group compared to the other groups (9).

In 2021, Martin et al*.* (9) added radiologic features of acute pancreatitis to the Connor criteria and found that 58% of patients with hyperamylasemia did not develop acute pancreatitis based on postoperative radiologic features. The researchers concluded that postoperative hyperamylasemia (POH) cannot be equated with POAP (35). The lack of analysis of the degree of residual pancreatic necrosis, a limitation of the Connor criteria, makes studies using this definition also lack a relevant clinical imaging diagnostic basis. The POAP cases in the Connor criteria group included many patients without radiologic features and clinical symptoms of pancreatitis.

Regardless of the diagnostic criteria, the high incidence of POAP deserves the attention of surgeons. In particular, the activation of pancreatic enzymes after POAP may lead to prolonged healing time or even non-healing of the pancreatic-intestinal anastomoses, resulting in postoperative pancreatic fistulas (POPFs), localized fluid accumulation in the abdominal cavity, infections, and even serious systemic complications (8, 9, 14, 35, 36). Some POAP may include acute necrotizing pancreatitis, which may pose serious hazards and even require secondary surgery for total pancreatectomy (10). Moreover, POAP is an independent risk factor of POPFs and DGE and may also increase the incidence of other serious complications and postoperative mortality (9, 27, 32, 34). Thus, prompt POAP diagnoses during the early postoperative period and treatment have the potential to improve patients' postoperative prognoses, reduce the incidence of more serious postoperative complications, and shorten the length of stay after pancreatic resections.

Some studies have suggested that the risk factors for acute pancreatitis after pancreatic resection include being a woman, not having received neoadjuvant therapy, and the presence of a soft pancreatic texture, a small main pancreatic duct diameter, or high C-reactive protein levels (14, 16, 17, 26, 37). We only included sex and the pancreatic texture as variables for our meta-analysis due to the small number of studies analyzing the other risk factors associated with POAP after PD. Our results showed that women had a higher risk of acute pancreatitis after PD than men, and patients with soft pancreatic textures (as judged intraoperatively by the surgeons) had a higher risk of POAP after surgery than patients with hard pancreatic textures. The heterogeneity in this respect that we observed may be due to differences in study diagnostic criteria and adjusted risk ratios in some of the included studies. A possible reason for woman being a risk factor for POAP is that woman have a higher body fat ratio which makes the pancreas softer. However, gender did not show a statistical difference in the multifactorial analysis in some of the studies reviewed in this systematic review, which were conducted without uniform confounding factors as well as classification methods. BMI, oncology, and neoadjuvant therapy were not included in some studies' multifactorial analysis, which may have led to biased results in some studies. Therefore, the conclusion that woman was a risk factor for POAP needs further validation. Moreover, a soft pancreatic texture has been recognized as an important risk factor for the development of pancreatic fistulas after pancreatic surgery (38, 39). A soft pancreatic texture used to be a sign of a small degree of pancreatic tissue fibrosis, even if the percentage of acinar cells or adipose tissue in the pancreas could not be determined without qualitative and quantitative histological analyses. Several studies have now reported results indicating a correlation between the density of acinar cells at the pancreatic cut edge and the occurrence of pancreatitis and postoperative pancreatic fistula after pancreatectomy (27, 40, 41).

The mechanisms of acute pancreatitis after pancreatic resection surgery are unclear. The possible factors that trigger its appearance or aggravation are direct injury to the pancreatic tissues during surgery (such as clamping of pancreatic tissue, dissection of pancreatic tissues, or reconstruction operation of pancreatic-intestinal anastomosis); pancreatic tissue ischemia caused by pulling, clamping, or dissection of surrounding vessels; use of drugs during anesthesia; and intraoperative/postoperative hypoxia or unstable blood pressure (37, 42–44). Maintaining perioperative vital signs as stable as possible, reducing intraoperative clamping of pancreatic tissues and surrounding vessels, and reducing unnecessary pancreatic tissue suturing operations seem to be important to prevent POAP.

### Strengths and limitations

The main strength of this study is that, to the best of our knowledge, it is the first meta-analysis of the incidence and risk factors of POAP after PD. Also, we analyzed the incidence and risk factors in subgroups according to the diagnostic criteria of POAP. Finally, the studies we included were from different countries, and most had adequate sample sizes. However, we are also aware of our study's limitations. The type of disease requiring PD surgery varied across studies, and we could not analyze those as a subgroup in this meta-analysis. In addition, we were not able to include some risk factors in the analysis because of the different definitions of POAP and the small number of relevant studies we found. Moreover, all the studies included were in English, and the lack of studies published in other languages and local journals may have introduced biases. Finally, we found publication bias in our meta-analysis, possibly because studies with positive results were more likely to be published than others.

In conclusion, we found that POAP is common after PD surgery. Differences in the incidence of POAP are due to the use of different diagnostic criteria, and being a woman and presenting a soft pancreas texture during surgery are risk factors for POAP. However, the current studies are few and limited to retrospective analyses; more prospective multicenter studies with large populations and uniform criteria are still needed to strengthen the analysis of POAP.

## Data Availability

The original contributions presented in the study are included in the article/**Supplementary Material**, further inquiries can be directed to the corresponding author.

## References

[1] Xu X Zheng C Zhao Y Chen W Huang Y. Enhanced recovery after surgery for pancreaticoduodenectomy: review of current evidence and trends. Int J Surg. (2018) 50:79–86. 10.1016/j.ijsu.2017.10.067 29081374

[2] Karim SAM Abdulla KS Abdulkarim QH Rahim FH. The outcomes and complications of pancreaticoduodenectomy (whipple procedure): cross sectional study. Int J Surg. (2018) 52:383–7. 10.1016/j.ijsu.2018.01.041 29438817

[3] Wang M Li D Chen R Huang X Li J Liu Y Laparoscopic versus open pancreatoduodenectomy for pancreatic or periampullary tumours: a multicentre, open-label, randomised controlled trial. Lancet Gastroenterol Hepatol. (2021) 6:438–47. 10.1016/S2468-1253(21)00054-6 33915091

[4] Dumonceau J-M Kapral C Aabakken L Papanikolaou IS Tringali A Vanbiervliet G ERCP-related adverse events: european society of gastrointestinal endoscopy (ESGE) guideline. Endoscopy. (2020) 52:127–49. 10.1055/a-1075-4080 31863440

[5] Ikenaga N Ohtsuka T Nakata K Watanabe Y Mori Y Nakamura M. Clinical significance of postoperative acute pancreatitis after pancreatoduodenectomy and distal pancreatectomy. Surgery. (2021) 169:732–7. 10.1016/j.surg.2020.06.040 32893007

[6] Haas GS Warshaw AL Daggett WM Aretz HT. Acute pancreatitis after cardiopulmonary bypass. Am J Surg. (1985) 149:508–15. 10.1016/s0002-9610(85)80048-9 2580453

[7] Feng F Tan H Li X Qiao Y Chen C Lin Y Incidence and risk factors of acute pancreatitis after scoliosis surgery: a prospective study. Spine (Phila Pa 1976). (2018) 43:630–6. 10.1097/BRS.0000000000002389 29016446

[8] Marchegiani G Barreto SG Bannone E Sarr M Vollmer CM Connor S Postpancreatectomy acute pancreatitis (PPAP): definition and grading from the international study group for pancreatic surgery (ISGPS). Ann Surg. (2022) 275:663–72. 10.1097/SLA.0000000000005226 34596077

[9] Connor S. Defining post-operative pancreatitis as a new pancreatic specific complication following pancreatic resection. HPB (Oxford). (2016) 18:642–51. 10.1016/j.hpb.2016.05.006 27485058 PMC4972444

[10] Globke B Timmermann L Klein F Fehrenbach U Pratschke J Bahra M Postoperative acute necrotizing pancreatitis of the pancreatic remnant (POANP): a new definition of severe pancreatitis following pancreaticoduodenectomy. HPB (Oxford). (2020) 22:445–51. 10.1016/j.hpb.2019.07.016 31431414

[11] Moher D Liberati A Tetzlaff J Altman DG. Preferred reporting items for systematic reviews and meta-analyses: the PRISMA statement. Br Med J. (2009) 339:b2535. 10.1371/journal.pmed.1000097 19622551 PMC2714657

[12] Page MJ McKenzie JE Bossuyt PM Boutron I Hoffmann TC Mulrow CD The PRISMA 2020 statement: an updated guideline for reporting systematic reviews. Br Med J. (2021) 372:n71. 10.1136/bmj.n71 33782057 PMC8005924

[13] Banks PA Bollen TL Dervenis C Gooszen HG Johnson CD Sarr MG Classification of acute pancreatitis—2012: revision of the Atlanta classification and definitions by international consensus. Gut. (2013) 62:102–11. 10.1136/gutjnl-2012-302779 23100216

[14] Wu S Wu H Xu G Zhao Y Xue F Dong S Risk factors and clinical impacts of post-pancreatectomy acute pancreatitis after pancreaticoduodenectomy: a single-center retrospective analysis of 298 patients based on the ISGPS definition and grading system. Front Surg. (2022) 9:916486. 10.3389/fsurg.2022.916486 35860201 PMC9289243

[15] Murakawa M Kamioka Y Kawahara S Yamamoto N Kobayashi S Ueno M Postoperative acute pancreatitis after pancreatic resection in patients with pancreatic ductal adenocarcinoma. Langenbecks Arch Surg. (2022) 407:1525–35. 10.1007/s00423-022-02481-0 35217927

[16] Chen H Wang C Shen Z Wang W Weng Y Ying X Post-pancreatectomy acute pancreatitis after pancreaticoduodenectomy: a distinct clinical entity. Ann Surg. (2022). 10.1097/SLA.0000000000005605. [Online ahead of print] 35848748

[17] Bonsdorff A Helanterä I Tarvainen T Sirén J Kokkola A Sallinen V. Prediction and consequences of postoperative pancreatitis after pancreaticoduodenectomy. BJS Open. (2022) 6:zrac012. 10.1093/bjsopen/zrac012 35470380 PMC9039121

[18] Bannone E Marchegiani G Perri G Procida G Vacca PG Cattelani A Postoperative serum hyperamylasemia (POH) predicts additional morbidity after pancreatoduodenectomy: it is not all about pancreatic fistula. Surgery. (2022) 172:715–22. 10.1016/j.surg.2022.04.003 35636983

[19] Yoo D Park SY Hwang DW Lee JH Song KB Lee W Lack of association between postoperative pancreatitis and other postoperative complications following pancreaticoduodenectomy. JCM. (2021) 10:1179. 10.3390/jcm10061179 33799863 PMC8001526

[20] Paik KY Oh JS Kim EK. Amylase level after pancreaticoduodenectomy in predicting postoperative pancreatic fistula. Asian J Surg. (2021) 44:636–40. 10.1016/j.asjsur.2020.11.022 33323317

[21] Doussot A Decrock M Calame P Georges P Turco C Lakkis Z Fluorescence-based pancreas stump perfusion is associated with postoperative acute pancreatitis after pancreatoduodenectomy a prospective cohort study. Pancreatology. (2021) 21:1023–9. 10.1016/j.pan.2021.05.009 34030965

[22] Ausania F Martínez-Pérez A Senra Del Rio P Borin A Melendez R Casal-Nuñez JE. Multifactorial mitigation strategy to reduce clinically relevant pancreatic fistula in high-risk pancreatojejunostomy following pancreaticoduodenectomy. Pancreatology. (2021) 21:466–72. 10.1016/j.pan.2020.12.019 33454209

[23] Partelli S Tamburrino D Andreasi V Mazzocato S Crippa S Perretti E Implications of increased serum amylase after pancreaticoduodenectomy: toward a better definition of clinically relevant postoperative acute pancreatitis. HPB (Oxford). (2020) 22:1645–53. 10.1016/j.hpb.2020.03.010 32291175

[24] Chen H Wang W Ying X Deng X Peng C Cheng D Predictive factors for postoperative pancreatitis after pancreaticoduodenectomy: a single-center retrospective analysis of 1465 patients. Pancreatology. (2020) 20:211–6. 10.1016/j.pan.2019.11.014 31831390

[25] Walsh RM Augustin T Aleassa EM Simon R El-Hayek KM Moslim MA Comparison of pancreas-sparing duodenectomy (PSD) and pancreatoduodenectomy (PD) for the management of duodenal polyposis syndromes. Surgery. (2019) 166:496–502. 10.1016/j.surg.2019.05.060 31474487

[26] Birgin E Reeg A Téoule P Rahbari NN Post S Reissfelder C Early postoperative pancreatitis following pancreaticoduodenectomy: what is clinically relevant postoperative pancreatitis? HPB (Oxford). (2019) 21:972–80. 10.1016/j.hpb.2018.11.006 30591305

[27] Nahm CB Brown KM Townend PJ Colvin E Howell VM Gill AJ Acinar cell density at the pancreatic resection margin is associated with post-pancreatectomy pancreatitis and the development of postoperative pancreatic fistula. HPB (Oxford). (2018) 20:432–40. 10.1016/j.hpb.2017.11.003 29307511

[28] Kühlbrey CM Samiei N Sick O Makowiec F Hopt UT Wittel UA. Pancreatitis after pancreatoduodenectomy predicts clinically relevant postoperative pancreatic Fistula. J Gastrointest Surg. (2017) 21:330–8. 10.1007/s11605-016-3305-x 27896656

[29] Jin S Shi X-J Wang S-Y Zhang P Lv G-Y Du X-H Drainage fluid and serum amylase levels accurately predict development of postoperative pancreatic fistula. World J Gastroenterol. (2017) 23:6357–64. 10.3748/wjg.v23.i34.6357 28974903 PMC5603503

[30] Renz BW Khalil PN Mikhailov M Graf S Schiergens TS Niess H Pancreaticoduodenectomy for adenocarcinoma of the pancreatic head is justified in elderly patients: a retrospective cohort study. Int J Surg. (2016) 28:118–25. 10.1016/j.ijsu.2016.02.064 26906329

[31] Dalla Valle R De Bellis M Pedrazzi G Lamecchi L Bianchi G Pellegrino C Can early serum lipase measurement be routinely implemented to rule out clinically significant pancreatic fistula after pancreaticoduodenectomy? Int J Surg. (2015) 21(Suppl 1):S50–54. 10.1016/j.ijsu.2015.04.090 26118616

[32] Weinberg L Wong D Karalapillai D Pearce B Tan CO Tay S The impact of fluid intervention on complications and length of hospital stay after pancreaticoduodenectomy (Whipple’s procedure). BMC Anesthesiol. (2014) 14:35. 10.1186/1471-2253-14-35 24839398 PMC4024015

[33] Makni A Bedioui H Jouini M Chebbi F Ksantini R Fetirich F Pancreaticojejunostomy vs. pancreaticogastrostomy following pancreaticoduodenectomy: results of comparative study. Minerva Chir. (2011) 66:295–302. 21873963

[34] Räty S Sand J Lantto E Nordback I. Postoperative acute pancreatitis as a major determinant of postoperative delayed gastric emptying after pancreaticoduodenectomy. J Gastrointest Surg. (2006) 10:1131–9. 10.1016/j.gassur.2006.05.012 16966032

[35] Loos M Strobel O Dietrich M Mehrabi A Ramouz A Al-Saeedi M Hyperamylasemia and acute pancreatitis after pancreatoduodenectomy: two different entities. Surgery. (2021) 169:369–76. 10.1016/j.surg.2020.07.050 32981689

[36] Loos M Strobel O Mehrabi A Mihaljevic AL Ramouz A Dietrich M Postoperative acute pancreatitis is a serious but rare complication after distal pancreatectomy. HPB (Oxford). (2021) 23:1339–48. 10.1016/j.hpb.2021.01.004 33546896

[37] Bannone E Andrianello S Marchegiani G Masini G Malleo G Bassi C Postoperative acute pancreatitis following pancreaticoduodenectomy: a determinant of Fistula potentially driven by the intraoperative fluid management. Ann Surg. (2018) 268:815–22. 10.1097/SLA.0000000000002900 30004917

[38] Sugimoto M Takahashi S Kobayashi T Kojima M Gotohda N Satake M Pancreatic perfusion data and post-pancreaticoduodenectomy outcomes. J Surg Res. (2015) 194:441–9. 10.1016/j.jss.2014.11.046 25541236

[39] Shen J Guo F Sun Y Zhao J Hu J Ke Z Predictive nomogram for postoperative pancreatic fistula following pancreaticoduodenectomy: a retrospective study. BMC Cancer. (2021) 21:550. 10.1186/s12885-021-08201-z 33992090 PMC8126152

[40] Laaninen M Bläuer M Vasama K Jin H Räty S Sand J The risk for immediate postoperative complications after pancreaticoduodenectomy is increased by high frequency of acinar cells and decreased by prevalent fibrosis of the cut edge of pancreas. Pancreas. (2012) 41:957–61. 10.1097/MPA.0b013e3182480b81 22699198

[41] Partelli S Andreasi V Schiavo Lena M Rancoita PMV Mazza M Mele S The role of acinar content at pancreatic resection margin in the development of postoperative pancreatic fistula and acute pancreatitis after pancreaticoduodenectomy. Surgery. (2021) 170:1215–22. 10.1016/j.surg.2021.03.047 33933282

[42] Ikenaga N Nakata K Fujita N Abe T Ideno N Ishigami K Clinical significance of postpancreatectomy acute pancreatitis defined by the international study group for pancreatic surgery. Ann Gastroenterol Surg. (2022) 6(6):842–50. 10.1002/ags3.12587 36338587 PMC9628230

[43] Lämsä T Jin H-T Nordback PH Sand J Luukkaala T Nordback I. Pancreatic injury response is different depending on the method of resecting the parenchyma. J Surg Res. (2009) 154:203–11. 10.1016/j.jss.2008.08.018 19394638

[44] Lämsä T Jin H-T Nordback PH Sand J Nordback I. Effects of diameter, number and tightness of sutures on pancreatic injury response. Dig Surg. (2008) 25:269–77. 10.1159/000135604 18628627

[45] Joliat G-R Petermann D Demartines N Schäfer M. Prediction of complications after pancreaticoduodenectomy: validation of a postoperative complication score. Pancreas. (2015) 44:1323–8. 10.1097/MPA.0000000000000399 26465955

