# Supplementary material for: Incidence and risk factors of postoperative acute pancreatitis after pancreaticoduodenectomy: a systematic review and meta-analysis
Source: Front Surg. 2023 May 9;10:1150053. doi: 10.3389/fsurg.2023.1150053 (PMC10203505; doi:10.3389/fsurg.2023.1150053)
Supplement: Supplementary file 1 [file Datasheet1.doc]

Supplementary Material

# Article Title Incidence and risk factors of postoperative acute pancreatitis after pancreaticoduodenectomy: A systematic review and meta-analysis

Zhouyu Wu, Kezhen Zong, Baoyong Zhou, Kunli Yin, Ming Li*, Anlan Zhang

*** Correspondence:** Ming Li

[liming@hospital.cqmu.edu.cn](mailto:liming@hospital.cqmu.edu.cn)

1. **Supplementary Search Strategy**

**PubMed** **Search Strategy**

(("Pancreaticoduodenectomy"[Mesh]) OR ((((((((((Pancreaticoduodenectomies[Title/Abstract]) OR (Pancreatoduodenectomy[Title/Abstract])) OR (Pancreatoduodenectomies[Title/Abstract])) OR (Duodenopancreatectomy[Title/Abstract])) OR (Duodenopancreatectomies[Title/Abstract])) OR (PPPD[Title/Abstract])) OR (Kausch-Whipple[Title/Abstract])) OR (Whipple*[Title/Abstract])) OR (ppWhipple*[Title/Abstract])) OR (pancreatic head resection[Title/Abstract]))) AND (("Pancreatitis"[Mesh]) OR (((((((((((((((((((Pancreatitis, Acute Edematous[Title/Abstract]) OR (Acute Edematous Pancreatitides[Title/Abstract])) OR (Edematous Pancreatitides, Acute[Title/Abstract])) OR (Edematous Pancreatitis, Acute[Title/Abstract])) OR (Pancreatitides, Acute Edematous[Title/Abstract])) OR (Acute Edematous Pancreatitis[Title/Abstract])) OR (Pancreatic Parenchymal Edema[Title/Abstract])) OR (Edema, Pancreatic Parenchymal[Title/Abstract])) OR (Pancreatic Parenchymal Edemas[Title/Abstract])) OR (Parenchymal Edema, Pancreatic[Title/Abstract])) OR (Pancreatic Parenchyma with Edema[Title/Abstract])) OR (Pancreatitis, Acute[Title/Abstract])) OR (Acute Pancreatitis[Title/Abstract])) OR (Acute Pancreatitides[Title/Abstract])) OR (Pancreatitides, Acute[Title/Abstract])) OR (Peripancreatic Fat Necrosis[Title/Abstract])) OR (Fat Necrosis, Peripancreatic[Title/Abstract])) OR (Necrosis, Peripancreatic Fat[Title/Abstract])) OR (Peripancreatic Fat Necroses[Title/Abstract]))) AND ((humans[Filter]) AND (english[Filter]))

**Web of science Search Strategy**

#1 TS=(Pancreaticoduodenectomy OR Pancreaticoduodenectomies OR Pancreatoduodenectomy OR Pancreatoduodenectomies OR Duodenopancreatectomy OR Duodenopancreatectomies OR PPPD OR Kausch-Whipple OR Whipple* OR ppWhipple* OR pancreatic head resection)

#2 TS=( Pancreatitis OR Pancreatitis, Acute Edematous OR Acute Edematous Pancreatitides OR Edematous Pancreatitides, Acute OR Edematous Pancreatitis, Acute OR Pancreatitides, Acute Edematous OR Acute Edematous Pancreatitis OR Pancreatic Parenchymal Edema OR Edema, Pancreatic Parenchymal OR Pancreatic Parenchymal Edemas OR Parenchymal Edema, Pancreatic OR Pancreatic Parenchyma with Edema OR Pancreatitis, Acute OR Acute Pancreatitis OR Acute Pancreatitides OR Pancreatitides, Acute OR Peripancreatic Fat Necrosis OR Fat Necrosis, Peripancreatic OR Necrosis, Peripancreatic Fat OR Peripancreatic Fat Necroses)

#1 AND #2

**Cochrane Library Search Strategy**

#1 Pancreatoduodenectomy

#2 Duodenopancreatectomies

#3 Pancreaticoduodenectomies

#4 Duodenopancreatectomy

#5 Pancreatoduodenectomies

#6 PPPD

#7 Kausch-Whipple

#8 Whipple*

#9 ppWhipple*

#10 pancreatic head resection

#11 MeSH descriptor: [Pancreaticoduodenectomy] explode all trees

#12 #11 OR #1 OR #2 OR #3 OR #4 OR #5 OR #6 OR #7 OR #8 OR #9 OR #10

#13 MeSH descriptor: [Pancreatitis] explode all trees

#14 Fat Necrosis, Peripancreatic

#15 Peripancreatic Fat Necrosis

#16 Peripancreatic Fat Necroses

#17 Necrosis, Peripancreatic Fat

#18 Pancreatitides, Acute Edematous

#19 Pancreatitis, Acute Edematous

#20 Edematous Pancreatitides, Acute

#21 Edematous Pancreatitis, Acute

#22 Acute Edematous Pancreatitides

#23 Acute Edematous Pancreatitis

#24 Pancreatic Parenchyma with Edema

#25 Edema, Pancreatic Parenchymal

#26 Parenchymal Edema, Pancreatic

#27 Pancreatic Parenchymal Edema

#28 Pancreatic Parenchymal Edemas

#29 Pancreatitides, Acute

#30 Acute Pancreatitis

#31 Pancreatitis, Acute

#32 Acute Pancreatitides

#33 #13 OR #14 OR #15 OR #16 OR #17 OR #18 OR #19 OR #20 OR #21 OR #22 OR #23 OR #24 OR #25 OR #26 OR #27 OR #28 OR #29 OR #30 OR #31 OR #32

#34 #12 AND #33

**Embase Search Strategy**

#8 #5 AND #6 AND [english]/lim AND [humans]/lim

#7 #5 AND #6

#6 #2 OR #4

#5 #1 OR #3

#4 'hereditary pancreatitis':ab,ti OR 'pancreas inflammation':ab,ti OR 'pancreatic inflammation':ab,ti OR 'traumatic pancreatitis':ab,ti

#3 'brunschwig operation':ab,ti OR duodenopancreatectomy:ab,ti OR 'pancreatico duodenectomy':ab,ti OR 'pancreato duodenal resection':ab,ti OR 'pancreato duodenectomy':ab,ti OR 'pancreatoduodenal resection':ab,ti OR pancreatoduodenectomy:ab,ti OR 'total pancreatic duodenectomy':ab,ti OR 'whipple operation':ab,ti OR 'whipple procedure':ab,ti OR 'whipple resection':ab,ti

#2 'pancreatitis'/exp

#1 'pancreaticoduodenectomy'/exp

1. **Supplementary Figures**


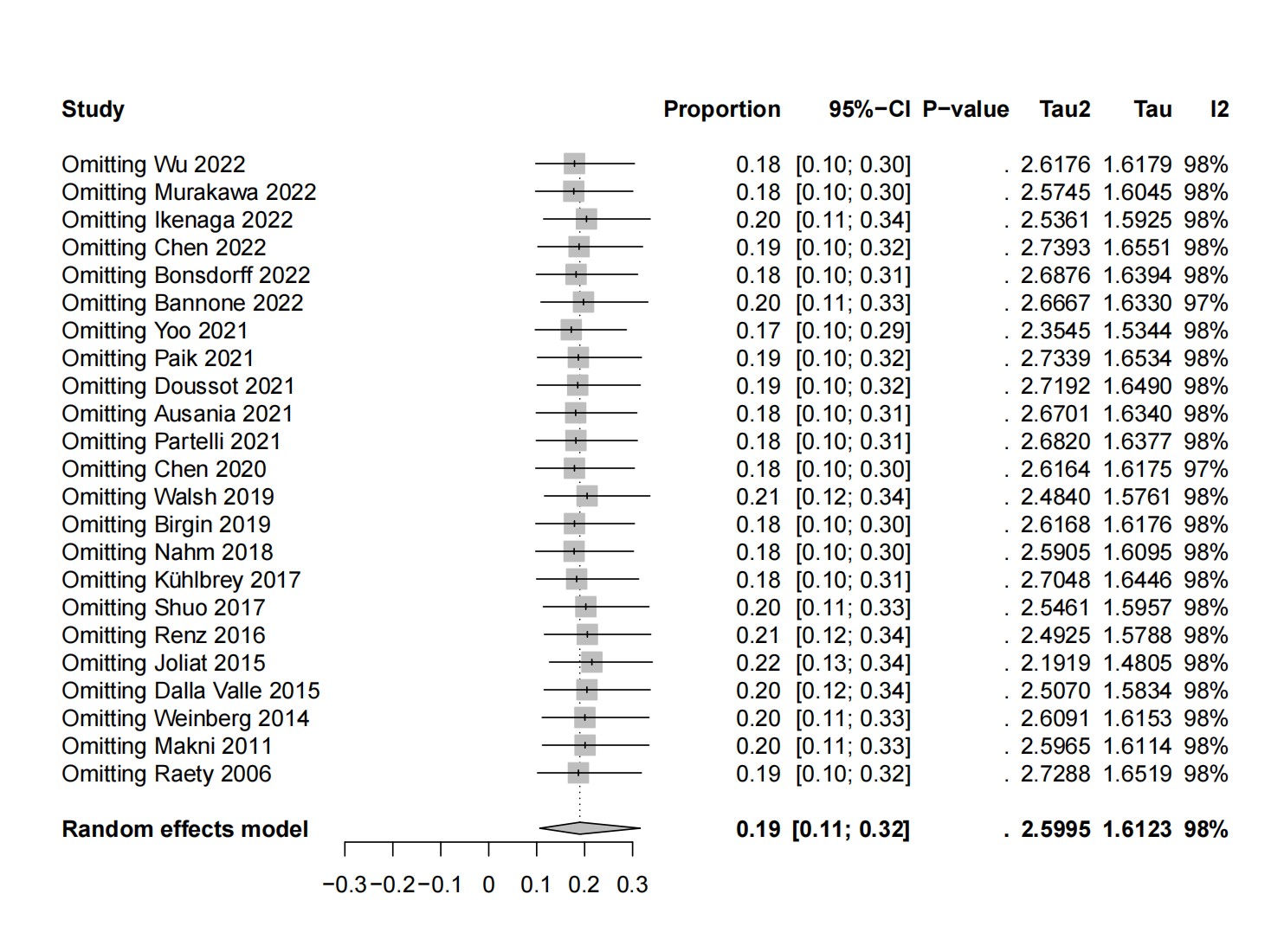


**Fig.1.** **Sensitivity analysis of POAP incidence**


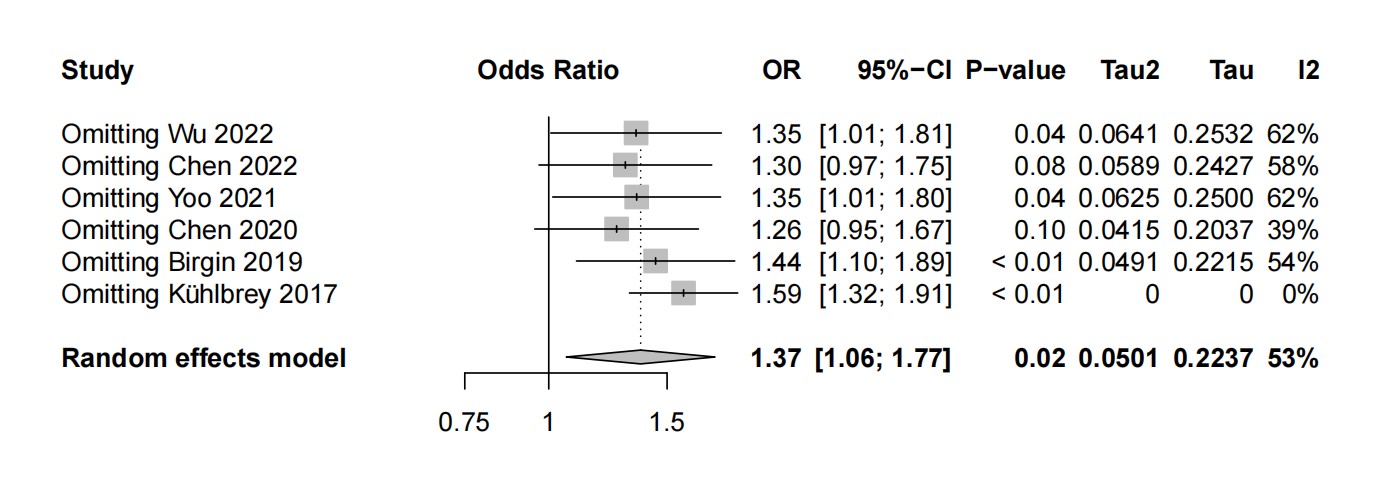


**Fig.2.** **Sensitivity analysis of sex**


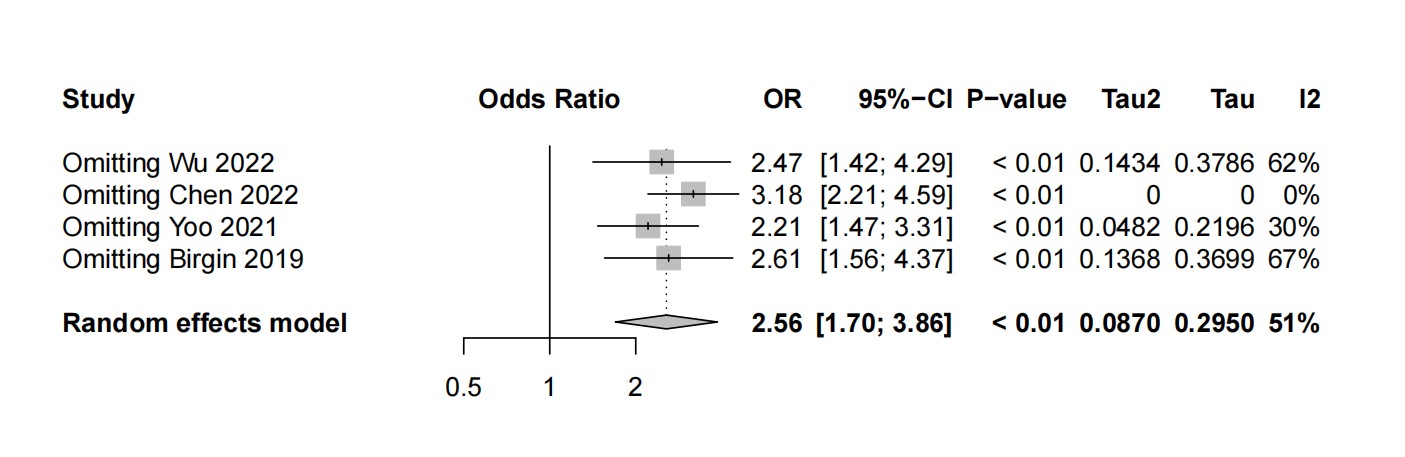


**Fig.3.** **Sensitivity analysis of pancreatic texture**
